# Supplementary material for: Bayesian refinement of protein structures and ensembles against SAXS data using molecular dynamics
Source: PLoS Comput Biol. 2017 Oct 18;13(10):e1005800. doi: 10.1371/journal.pcbi.1005800 (PMC5662244; doi:10.1371/journal.pcbi.1005800)
Supplement: S1 Archive — (BZ2) [file pcbi.1005800.s011.tar.bz2 › gromacs_4.62_bayesian_saxs_refi/share/html/online/flow.html]

Flow Chart


| GROMACS Flow chart Main Table of Contents |

**VERSION 4.6  
Sat 19 Jan 2013**

---

This is a flow chart of a typical GROMACS MD run of a protein
in a box of water.
A more detailed example is available in the
Getting Started
section. Several steps of energy minimization may be necessary,
these consist of cycles: grompp -> mdrun.

|  |  |  |  |  |  |  |  |  |
| --- | --- | --- | --- | --- | --- | --- | --- | --- |
| eiwit.pdb | |  |  |  |  |  |  |  |
| Generate a GROMACS topology | |  | **pdb2gmx** | | |  |  |  |
|  |  |  |  |  |  |  |  |  |
|  |  |  | conf.gro |  | topol.top |  |  |  |
|  |  |  |  |  |  |  |  |  |
| Enlarge the box | |  | **editconf** |  |  |  |  |  |
|  |  |  |  |  |  |  |  |  |
|  |  |  | conf.gro |  |  |  |  |  |
|  |  |  |  |  |  |  |  |  |
| Solvate protein | |  | **genbox** | | |  |  |  |
|  |  |  |  |  |  |  |  |  |
|  |  |  | conf.gro |  | topol.top |  |  |  |
| grompp.mdp | |  |  |  |  |  |  |  |
| Generate mdrun input file | |  | **grompp** | | |  |  |  |
|  |  |  |  |  |  | Continuation |  |  |
|  | |  | topol.tpr | | | state.cpt |  |  |
|  |  |  |  |  |  |  |  |  |
| Run the simulation (EM or MD) | |  | **mdrun** | | |  |  |  |
|  |  |  |  |  |  |  |  |  |
|  |  |  | traj.xtc / traj.trr |  | ener.edr |  |  |  |
|  |  |  |  |  |  |  |  |  |
| Analysis | |  | **g\_...**     **ngmx** |  | **g\_energy** |  |  |  |

  


---

http://www.gromacs.org
